# Sex differences in risk factors of uncomplicated colonic diverticulosis in a metropolitan area from Northern China

**DOI:** 10.1038/s41598-017-18517-1

**Published:** 2018-01-09

**Authors:** Fang Yang, Yanmin Zheng, Xihui Jiang, Zhengyan Su, Ya Wang, Lin Lin, Houning Lv, Jie Zhang, Jingwen Zhao, Bangmao Wang, Kui Jiang, Chao Sun

**Affiliations:** 1 Department of Gastroenterology and Hepatology, Tianjin Medical University General Hospital, Anshan Road 154, Heping District, Tianjin 300052 China; 2 Tianjin Institute of Digestive Disease, Tianjin Medical University General Hospital, Anshan Road 154, Heping District, Tianjin 300052 China

## Abstract

As the world’s most populated and rapidly aging country, there is limited information on sex-related differences in factors regarding uncomplicated colonic diverticulosis in China. We aimed to investigate sex differences in individual risk factor in a northern metropolis. Patients with colonic diverticulosis who underwent indicated colonoscopy were queried with respect to medical history and demographic features. Demographic information, life style factors and co-morbidities were retrieved from a prospective dataset. Multiple regression analyses were performed to determine precipitating factors of diverticula. Of 4,386 enrolled patients, colonic diverticulosis were detected in 218 cases (4.97%). Multiple logistic regression analysis implicated increasing age (OR = 1.05, 95%CI 1.03–1.06, P < 0.001), red meat ≥100 g/d (OR = 2.53, 95%CI 1.72–3.70, P < 0.001), smoking (OR = 2.14, 95%CI 1.05–4.33, P = 0.035), rheumatologic diseases (OR = 3.38, 95%CI 1.09–10.5, P = 0.035) and NSAIDs (OR = 2.11, 95%CI 1.12–3.97, P = 0.020) were significantly associated with diverticulosis in men, whilst advancing age (OR = 1.03, 95%CI 1.01–1.05, P = 0.013), BMI (OR = 1.12, 95%CI 1.04–1.19, P = 0.001), smoking (OR = 10.2, 95%CI 2.81–37.4, P < 0.001), rheumatologic diseases (OR = 8.04, 95%CI 3.05–21.2, P < 0.001), hypertension (OR = 1.76, 95%CI 1.01–3.06, P = 0.047), colonic polyps (OR = 3.12, 95%CI 1.82–5.36, P < 0.001) and antihypertensive medications (OR = 2.99, 95%CI 1.66–5.39, P < 0.001) in women. In conclusion, it is pivotal to take account of differentially sex-related factors in regard to the development of uncomplicated colonic diverticulosis.

## Introduction

China has underwent rapid growth of aging population during recent decades; those aged ≥ 75 years accounted for 3.5% of the population in 2013, and there were 200 million elderly residents aged ≥ 65 years in 2014^1^. Therefore the burden of diseases, especially entity with a higher prevalence among the elderly, including uncomplicated colonic diverticulosis, is predicted to increase in the future^2,3^. As a matter of fact, the rates of diverticula have dramatically increased from 2.78% in 2011 to 4.98% in 2015 according to dataset from our endoscopy center (data not shown). Aside from aging, the pathogenesis of diverticulosis is thought to be multifactorial, including diet, colonic motility, obesity, medications and genetic factors^4^. More recently, it has been recognized that altered gut microbiota composition and abnormal immune signatures may contribute to the development and progression of uncomplicated diverticular disease^5,6^. Of note, explicit sex-specific diet-microbiota correlations have been documented in humans^7^. After reviewing current body of literature, gender was evidently associated with the presence of diverticulosis^5,8–10^. Thus, it is plausible to hypothesize that sex-related differences regarding risk factors for the formation of diverticula. Collectively, we aimed to assess potential factors related to diverticulosis by capturing and analyzing demographic, environmental and pathological information on recruited subjects on a sex disparity basis.

## Results

### Prevalence and sex differences in baseline characteristics

**Figure 1:**
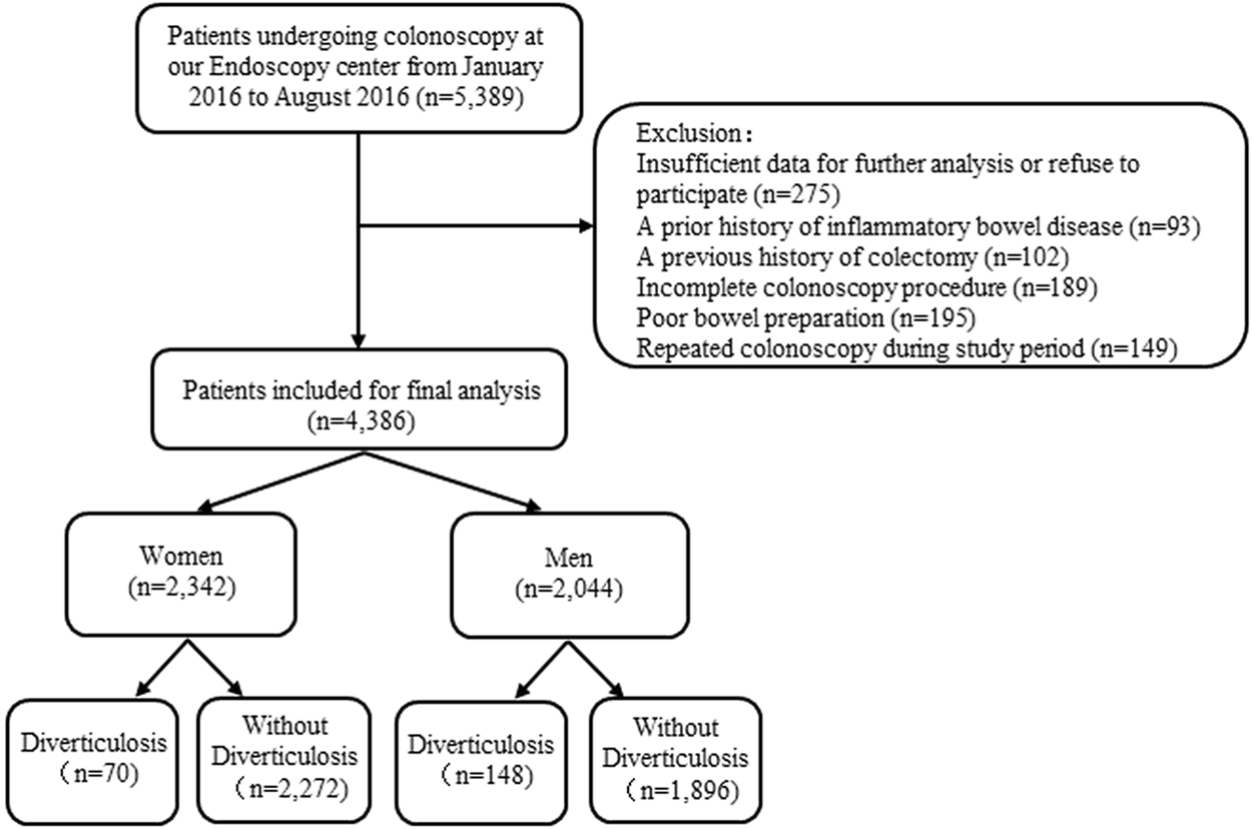
Flow diagram of patients included for current study.

A total of 4,386 consecutive cases undergoing complete colonoscopy were finally recruited during this study period (Fig. 1). Of these, 218 participants with uncomplicated colonic diverticulosis were finally identified, comprising 148 men (7.24% of 2,044 men) and 70 women (2.99% of 2,342 women). The overall prevalence of colonic diverticulosis was 4.97%. Diverticula were right-sided in 67.0%, bilateral in 18.3%, and left-sided in 14.7% of patients with diverticulosis.

Of the entire population, women were more likely than men to be older (mean age of 54.7 years *vs* 52.6 years, *P* < 0.001). There were more women aged 50–59 years (33.5% *vs* 23.9%) and 60–69 years (32.2% *vs* 29.2%) in comparison with men. Moreover, women had lower levels of BMI and lower frequencies of weekly exercise, red meat, smoking status as well as alcoholic consumption (*P* < 0.001 for all). Concerning comorbidities and medication, the prevalence of colonic polyps was significant higher in men, whereas the use of mucosal protective drugs was more common in women (Table 1).

**Table 1:** Sex differences in baseline characteristics of the study population.

Characteristics	Men (n = 2,044)	Women (n = 2,342)	*P* values	Cohen’s D
**Demographics and life style factors**				
Age (years), mean (SD)	52.6 (14.4)	54.7 (12.9)	<**0**.**001**	0.15
Age (years), n (%)			<**0**.**001**	0.29
≤39	460 (22.5)	319 (13.6)		
40–49	303 (14.8)	289 (12.4)		
50–59	489 (23.9)	784 (33.5)		
60–69	597 (29.2)	755 (32.2)		
≥70	195 (9.6)	195 (8.3)		
Education (years), n (%)			0.999	0.00
≤6	172 (8.4)	197 (8.4)		
07-Sep	496 (24.3)	570 (24.3)		
10-Dec	516 (25.2)	592 (25.3)		
>12	860 (42.1)	983 (42.0)		
Residence, n (%)			0.870	0.01
Urban	1,649 (80.7)	1,894 (80.9)		
Rural	395 (19.3)	448 (19.1)		
BMI (kg/m^2^), mean (SD)	24.2 (3.1)	23.0 (3.4)	<**0**.**001**	0.37
BMI, n (%)			**<0**.**001**	0.31
<25	1,313 (64.2)	1,834 (78.3)		
25–30	649 (31.8)	412 (17.6)		
>30	82 (4.0)	96 (4.1)		
Exercise habit, n (%)			<0.001	0.26
≤3 times/week	857 (41.9)	1,281 (54.7)		
>3 times/week	1,187 (58.1)	1,061 (45.3)		
Red Meat, n (%)			**<0**.**001**	0.32
<100 g/d	1,594 (78.0)	2,138 (91.3)		
≥100 g/d	450 (22.0)	204 (8.7)		
Smoking index, n (%)			**<0**.**001**	0.85
Nonsmoker	1,162 (56.8)	2,314 (98.8)		
<400	294 (14.4)	12 (0.5)		
≥400	588 (28.8)	16 (0.7)		
Alcohol consumption, n (%)			**<0**.**001**	0.54
Non-drinker	1,548 (75.7)	2,315 (98.8)		
Light/Moderate drinker (1–350 g/week)	96 (4.7)	27 (1.2)		
Heavy drinker (≥351 g/week)	400 (19.6)	0 (0)		
**Comorbidities**, **n** (**%**)				
Hypertension	371 (18.2)	428 (18.3)	0.915	0.00
Diabetes mellitus	151 (7.4)	143 (6.1)	0.09	0.05
Coronary heart disease	94 (4.6)	111 (4.7)	0.826	0.00
Colonic polyps	1,030 (50.4)	919 (39.2)	<**0**.**001**	0.22
Upper gastrointestinal diseases^a^	151 (7.4)	164 (7.0)	0.622	0.02
Hepato-biliary diseases^b^	92 (4.5)	86 (3.7)	0.165	0.04
Dyslipidemia	46 (2.3)	64 (2.7)	0.308	0.03
Rheumatologic diseases^c^	46 (2.3)	43 (1.8)	0.331	0.03
Miscellaneous^d^	186 (9.1)	203 (8.7)	0.616	0.01
**Medication**, **n** (**%**)				
NSAIDs	115 (5.6)	125 (5.3)	0.675	0.01
Corticosteroid	25 (1.2)	32 (1.4)	0.676	0.02
PPIs	123 (6.0)	169 (7.2)	0.112	0.05
Mucosal protective drugs	432 (21.1)	607 (25.9)	<**0**.**001**	0.12
Antihypertension drugs	363 (17.8)	422 (18.0)	0.823	0.01
Hypoglycemic medications	149 (7.3)	138 (5.9)	0.62	0.05

Data are expressed as mean ± SD or number (percentage).

*BMI* body mass index, *NSAIDs* non-steroidal anti-inflammatory drugs, *PPIs* proton pump inhibitors.

^a^Reflux esophagitis, atrophic gastritis, chronic gastritis, etc.

^b^Cholelithiasis and fatty liver disease.

^c^Rheumatoid arthritis and gout.

^d^Osteoporosis, arrhythmia, cerebral infarction, thyroid diseases, prostate diseases, etc.

### Sex differences in risk factors for diverticulosis

We first implicated that male gender was a precipitating factor for the presence of diverticulosis in whole study population (Table S1). In addition, concerning dramatically disparities regarding the baseline features, we next investigated risk factors associated with the formation of diverticula according to sex differences.

Table 2 implicated that age, smoking index, hypertension, rheumatologic diseases, antihypertension drugs and NSAIDs were associated with the presence of colonic diverticulosis in both men and women on the univariate analyses. Moreover, red meat was a precipitating factor for diverticula in men, whilst BMI and concomitant polyps were associated with the presence of colonic diverticulosis in women.

**Table 2:** The sex differences in risk factors of colonic diverticulosis on univariate analyses.

	Men			Women		
	Div (+) (n = 148)	Div (−) (n = 1,896)	*P* values	Div (+) (n = 70)	Div (−) (n = 2,272)	*P* values
Age, (years), mean (SD)	59.5 (10.6)	52 (14.5)	**<0.001**	58.0 (11.8)	54.6 (12.9)	**0.029**
Age (years), n (%)			**<0.001**			**<0.001**
≤39	6 (4.0)	285 (15.0)		10 (14.3)	309 (13.6)	
40–49	18 (12.2)	440 (23.2)		6 (8.6)	283 (12.5)	
50–59	49 (33.1)	548 (28.9)		12 (17.1)	772 (33.9)	
60–69	49 (33.1)	169 (8.9)		24 (34.3)	731(32.2)	
≥70	26 (17.6)	454 (24.0)		18 (25.7)	177 (7.8)	
Education (years), n (%)			0.971			0.999
≤6	14 (9.5)	158 (8.3)		6 (8.6)	191 (8.4)	
7–9	35 (23.6)	461 (24.3)		17 (24.3)	553 (24.3)	
10–Dec	37 (25.0)	479 (25.3)		18 (25.7)	574 (25.3)	
>12	62 (41.9)	798 (42.1)		29 (41.4)	954 (42.0)	
Residence, n (%)			0.243			0.619
Urban	114 (77.0)	1,535 (81.0)		55 (78.6)	1,839 (80.9)	
Rural	34 (23.0)	361 (19.0)		15 (21.4)	433 (19.1)	
BMI (kg/m^2^), mean (SD)	24.6 (3.4)	24.2 (3.1)	0.105	24.5 (3.8)	22.9 (3.3)	**<0.001**
BMI, n (%)			0.105			**<0.001**
<25	86 (58.1)	1,227 (64.7)		41 (58.6)	1,793 (78.9)	
25–30	52 (35.1)	597 (31.5)		19 (27.1)	393 (17.3)	
>30	10 (6.8)	72 (3.8)		10 (14.3)	86 (3.8)	
Exercise habit, n (%)			0.483			0.181
≤3 times/week	58 (39.2)	799 (42.1)		44 (62.9)	1,237 (54.4)	
>3 times/week	90 (60.8)	1,097 (57.9)		26 (37.1)	1,035 (45.6)	
Red Meat, n (%)			**0.001**			0.83
<100 g/d	99 (66.9)	1,495 (78.9)		65 (92.9)	2,073 (91.2)	
≥100 g/d	49 (33.1)	401 (21.1)		5 (7.1)	199 (8.8)	
Smoking index, n (%)			**0.009**			**<0.001**
Nonsmoker	67 (45.3)	1,095 (57.8)		65 (92.8)	2,249 (99.0)	
<400	30 (20.3)	264 (13.9)		1 (1.4)	11 (0.5)	
≥400	51 (34.4)	537 (28.3)		4 (5.8)	12 (0.5)	
Alcohol consumption, n (%)			0.153			0.199
Non-drinker	121 (81.8)	1,427 (75.3)		70 (100)	2,245 (98.8)	
Light/moderate drinker	7 (4.7)	89 (4.7)		0 (0)	27 (1.2)	
(1–350 g/week)				0 (0)	0 (0)	
Heavy drinker (≥351 g/week)	20 (13.5)	380 (20.0)				
Hypertension	42 (28.4)	329 (17.4)	**0.002**	23 (32.9)	405 (17.8)	**0.003**
Diabetes mellitus	13 (8.8)	138 (7.3)	0.512	8 (11.4)	135 (5.9)	0.071
Coronary heart disease	10 (6.8)	84 (4.4)	0.217	5 (7.1)	106 (4.7)	0.381
Colonic polyps	81 (54.7)	949 (50.1)	0.306	42 (60.0)	877 (38.6)	**0.01**
Upper gastrointestinal diseases	12 (8.1)	139 (7.3)	0.728	5 (7.1)	159 (7.0)	0.963
Hepato-biliary diseases	5 (3.4)	87 (4.6)	0.494	5 (7.1)	81 (3.6)	0.117
Dyslipidemia	4 (2.7)	42 (2.2)	0.571	4 (5.7)	60 (2.6)	0.122
Rheumatologic diseases	9 (6.1)	37 (2.0)	**0.001**	10 (14.3)	33 (1.5)	**<0.001**
Miscellaneous	14 (9.5)	172 (9.1)	0.874	10 (14.3)	193 (8.5)	0.09
NSAIDs	24 (16.2)	91 (4.8)	**<0.001**	10 (14.3)	115 (5.1)	**0.003**
Corticosteroid	4 (2.7)	21 (1.1)	0.102	3 (4.3)	29 (1.3)	0.068
PPIs	11 (7.4)	112 (5.9)	0.47	5 (7.1)	164 (7.2)	1
Mucosal protective drugs	35 (23.6)	397 (20.9)	0.464	24 (34.3)	583 (25.7)	0.127
Antihypertension medications	40 (27.0)	323 (17.0)	**0.004**	22 (31.4)	400 (17.6)	**0.006**
Hypoglycemic medications	15 (10.0)	134 (7.1)	0.186	7 (10.0)	131 (5.8)	0.188

Data are expressed as mean ± SD or number (percentage).

Div (−): patients without diverticulosis.

Div (+): patients with diverticulosis.

*BMI* body mass index, *NSAIDs* non-steroidal anti-inflammatory drugs, *PPIs* proton pump inhibitors.

**Figure 2:**
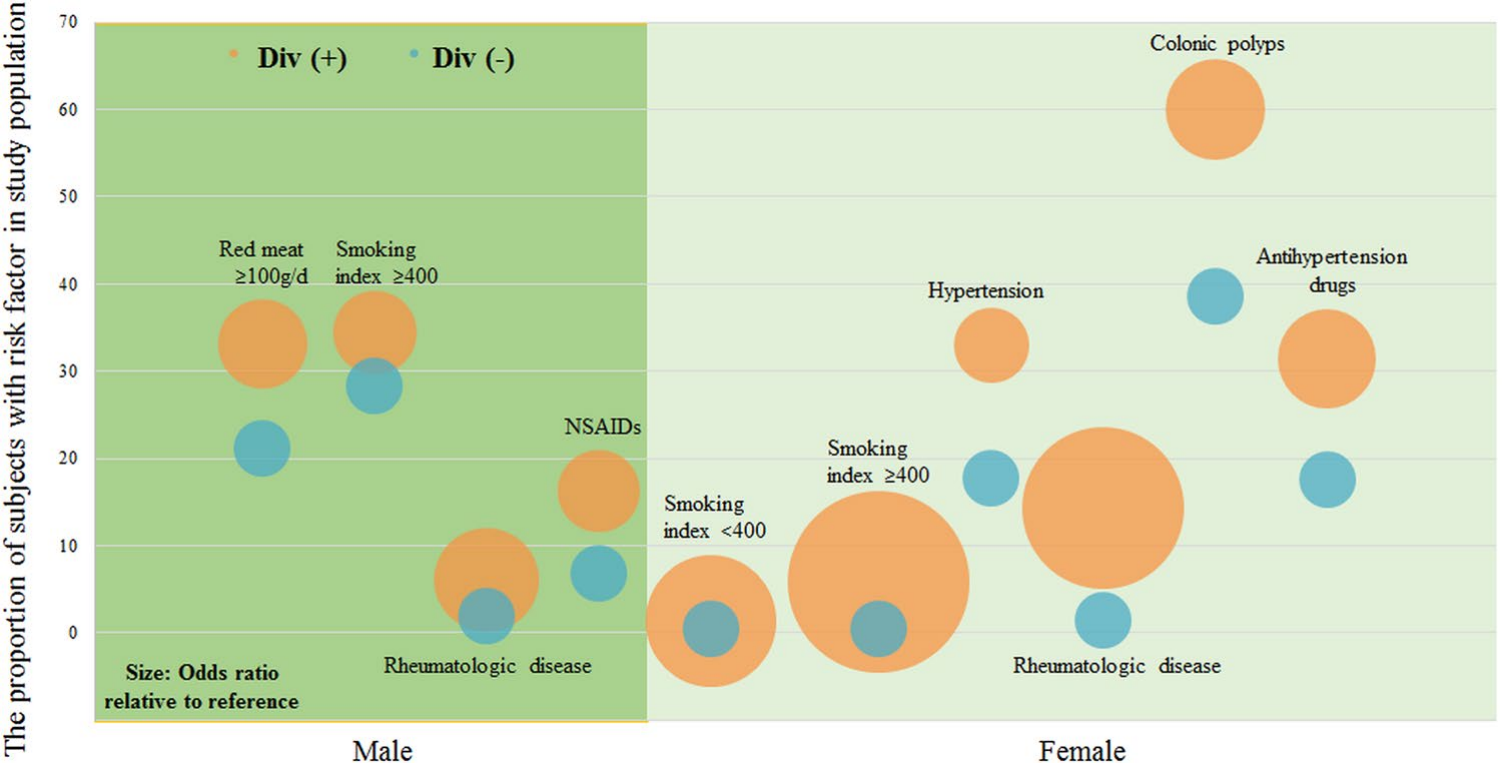
Proportion of subjects with indicated risk factor (*P* < 0.05 for all) and odds ratio (OR) relative to reference in study population by the presence of diverticula. *Circles* show the proportion (*y-axi*s) and OR (diameter) relative to each reference. Div (+), patients with diverticulosis; Div (−), patients without diverticulosis.

Based on multivariate analyses, advancing age, increasing smoking index and rheumatologic comorbidity were significantly associated with diverticulosis in both men and women. The risk of colonic diverticulosis increased with age; the corresponding adjusted OR (95% CI) were 1.05 (1.03–1.06) and 1.03 (1.01–1.05) in men and women, respectively. Moreover, individuals with smoking index no less than 400 and rheumatologic diseases had a higher risk of diverticulosis, with adjusted OR (95% CI) of 2.14 (1.05–4.33) and 10.2 (2.81–37.4) for smoking status, 3.38 (1.09–10.5) and 8.04 (3.05–21.2) for rheumatologic comorbidity in men and women, respectively. The corresponding adjusted ORs (95% CI) in men were 2.53 (1.72–3.70) for consumed red meat ≥ 100 g per day and 2.11 (1.12–3.97) for NSAIDs; in women, adjusted ORs (95% CI) were 1.12 (1.04–1.19) for elevated BMI, 1.76 (1.01–3.06) for hypertension, 3.12 (1.82–5.36) for polyps and 2.99 (1.66–5.39) for regular usage of antihypertensive medication (Table 3 and Fig. 2).

**Table 3:** The sex differences in risk factors of colonic diverticulosis on multivariate analyses.

Risk factors	Reference	*B*	Adjusted OR (95% CI)	*P* values
**Men**				
Age	—	0.047	1.05 (1.03, 1.06)	<**0**.**001**
Red meat ≥ 100 g/d	Red meat < 100 g/d	0.926	2.53 (1.72, 3.70)	**<0**.**001**
Smoking index				
<400	Nonsmoker	0.317	1.37 (0.96, 1.97)	0.084
≥400	Nonsmoker	0.759	2.14 (1.05, 4.33)	**0**.**035**
Hypertension	Non-hypertension	1.216	3.37 (0.69, 16.4)	0.133
Rheumatologic diseases	Non-rheumatologic diseases	1.216	3.38 (1.09, 10.5)	**0**.**035**
NSAIDs	Non-NSAIDs	0.748	2.11 (1.12, 3.97)	**0**.**020**
Antihypertension medications	Non-Antihypertension medications	0.447	1.08 (0.52, 11.8)	0.159
**Women**				
Age	—	0.028	1.03 (1.01, 1.05)	**0**.**013**
BMI (kg/m²)	—	0.109	1.12 (1.04, 1.19)	**0**.**001**
Smoking index				
<400	Nonsmoker	1.680	5.37 (1.03, 27.9)	**0**.**046**
≥400	Nonsmoker	2.327	10.2 (2.81, 37.4)	<**0**.**001**
Hypertension	Non-hypertension	0.564	1.76 (1.01, 3.06)	**0**.**047**
Rheumatologic diseases	Non-rheumatologic diseases	2.084	8.04 (3.05, 21.2)	<**0**.**001**
Colonic polyps	Non-colonic polyps	1.138	3.12 (1.82, 5.36)	<**0**.**001**
Antihypertension drugs	Non-antihypertension drugs	1.094	2.99 (1.66, 5.39)	<**0**.**001**
NSAIDs	Non-NSAIDs	0.095	1.10(0.46, 2.66)	0.833

*BMI* body mass index, *NSAIDs* non-steroidal anti-inflammatory drugs, *OR* odds ratio, *CI* confidence interval.

## Discussion

To our knowledge, this is the first cross-sectional study to evaluate relevant risk factors of uncomplicated colonic diverticulosis in mainland China. Furthermore, we reported sex differences in risk factors for the formation of diverticula, whereas older age, increasing tabacco consumption and rheumatologic diseases were independent risk factors in both men and women.

It has been renewed interest and urgent reappraisal towards colonic diverticula, as this entity and its related complications may impose a serious burden on public healthcare systems. With the growing body of elder population and advent of widely colonoscopy-based examinations, the estimated prevalence of the disease has been still increasing in Western countries on a yearly basis. While still low in comparison to Western countries, an increasing trend regarding prevalence was also observed in several Asian countries and areas, including Singapore (45%), Thailand (28.5%), Japan (20.3%) and Taiwan (13.5%)^10–13^. More recently, a study form Southern China has implied an overall diverticulosis prevalence as 1.97% without significant change over more than one decade^14^. However, the representative of study mentioned above is limited, partly due to relatively small proportion of elderly, various lifestyle and dietary pattern differences across North and South China^15^. Aside from aging, environmental factors have also been identified as risk factors for the formation of diverticula. Recent epidemiological data indicated that genetic variants and underlying molecular pathobiology may contribute to the development to some extent. Intriguingly, Barbara and colleagues provided evidence integrating mucosal immunological information with gut microbiota and metabolic profiles with respect to the pathogenesis of diverticulosis^6^. Diet and sex have been broadly investigated and suggested as possible predisposed factors for the presence of diverticula in extensive research. Diet, as a source of colonizing bacteria, can alter the gut nutritional environment and subsequently influence the composition of microbiota^7^. As being a genetic trait, sex, may also affect the microbiota through hormone-microbe interaction and sex-specific immune responses. It has been addressed that studies omitting sex difference will overlook these interactions and consequently fail to determine environmental impacts. Moreover, several observations suggested a sharp sex distinction in regard to diverticular disease and its manifestations, that is, men were predisposed to diverticular bleeding while women were more likely present with strictures/obstructions^16^. Collectively, we intend to detect sex-related risk factors of colonic diverticulosis in this cross-sectional study after reviewing dramatically distinct baseline characteristics within the sexes.

Although a growing body of literature has been published to demonstrate the association between diverticulosis and conventional risk factors, the results remain controversial and predominantly arise in theoretical models. Of note, it is evident that several long-held beliefs, time-honored concepts and classic teachings on diverticula are challenged by recent research. For instance, dietary fiber is no longer regarded as protective against the development of diverticulosis^3^.

We found advancing age, heavy tobacco consumption and rheumatologic diseases were strong risk factors associated with the formation of diverticular in both men and women. Age has been posited as the most pronounced risk factor for diverticulosis in previous many reports. It has been proposed that elderly may develop abnormal thickness of colonic wall and frail muscular structure which is susceptible to incremental intraluminal pressure^17,18^. A positive association of diverticulosis and smoking status was unveiled in our study, whose role and exact mechanism were still in controversy; however, pertaining to a proinflammatory stimulus, disturbance on colonic motility and impacts against microflora homeostasis have all been postulated to play a role^19–21^. Furthermore, we also noted a higher prevalence of rheumatologic disorders in patients with diverticulosis. This is in accordance with previous findings implying association between inflammatory connective tissue diseases and diverticula^5,22^. While, smoking by itself is so far the most well-established environmental risk factor in the development of rheumatoid arthritis^23^.

In women, we also demonstrated that concomitant hypertension and antihypertensive medications were more prevalent in subjects with diverticulosis, which is consistent with previous findings by Yazici and colleagues^5^. This may partly be a reflection of older age amongst patients with diverticula, and elder women were more likely to have hypertension and higher systolic blood pressure than men^24^. Additionally, an outperformed study in China implied that a traditional northern dietary pattern was closely associated with both higher prevalence of hypertension and blood pressure, and the associations were largely explained by BMI. It is noteworthy that women with diverticulosis were likely to be obese (BMI > 30) compared to men as shown in Table 2. Moreover, numerous studies have been conducted in investigating the effects of obesity on shifts in microbiota in human beings, and further efforts are warranted to see if it can explain increasing risk of diverticulosis in such population^25–27^. At last, the coexisting colonic polyps and diverticulosis should be attributed to many similarities form the epidemiologic stand points and risk factors instead of causative relationship^28,29^.

Our results indicated that higher intake of red meat consumption and the usage of NSAIDs were both associated with the presence of diverticula in men. Whether the association between red meat consumption and uncomplicated diverticular disease remains unclear, red meat intake might influence the gut microflora or contribute to high energy intakes, which provide partial explanation for these correlations^23^. NSAIDs users hold more risk to develop symptomatic diverticular diseases than non-users. And it was hypothesized that this increased risk was attributable to mucosal damage resulting in impaired barrier of the colonic mucosa allowing bacteria translocation, which aggravate inflammation^30^.

We should not overlook potential limitations of our study. First, it is difficult to formally draw conclusions about causality due to its cross-sectional design in nature. However, this problem is mitigated by the inclusion of subjects with various colonoscopic indications, although residual bias may still exist. Second, data on lifestyle factors were retrieved by questionnaires referring to a period of almost one year, which may give rise to recall error. However, a face-to-face reviewed system with two well trained investigators (FY and YZ) can eliminate this adverse impact to some degree. Third, we lack of detailed information on dietary patterns (e.g. fruit, bean or nut fiber). Although we tried to adjust for major demographic and environmental factors simultaneously, residual confounding by unmeasured or unknown factors might be present. Conversely, taken account of the magnitude of many of the risk estimates and consistency of our results with past reports in regard to risk factors of colonic diverticula, it is improbable that all of the identified risk differences are attributable to the results of residual confounding. Fourth, we failed to determine a definite association between alcohol use and the presence of diverticulosis, inconsistent with prior cohort studies, while influence of this upon diverticulosis is still controversial^3,13,20,31^. Our sample population had therefore a higher proportion of non-drinkers in both genders (more than 75% of enrolled participants) compared with research from western countries which have made it difficult to detect an association between alcohol and diverticulosis. Actually, the East-West paradox are paramount in disease prevalence and phenotype^28^.

## Conclusions

In conclusion, the prevalence of diverticulosis was 7.24% in men and 2.99% in women among the study population in a metropolitan area from Northern China. This study was the first to explore sex differences on associated risk factors among patients with uncomplicated colonic diverticulosis. Advancing age, increasing smoking index and rheumatologic comorbidity were significantly associated with diverticulosis in both men and women. Specifically, men with higher red meat consumption and use of NSAIDs were predisposed to colonic diverticula, whilst this entity was more prevalent in women with incremental BMI, concurrent hypertension, polyps and use of antihypertension drugs. Therefore, our results pave the way for further large-scale survey aiming at identifying sex-specific conventional risk factors; individualized uncomplicated diverticulosis prevention/intervention and promotion of healthy lifestyle are critical.

## Materials and Methods

### Participants and study design

We conducted a cross-sectional single-center study in adults undergoing colonoscopy at the Endoscopy Center, Department of Gastroenterology and Hepatology, Tianjin Medical University General Hospital (TJMUGH) from January 2016 to August 2016. The TGMUGH is a tertiary referral hospital with 2,468 beds located in Tianjin - a metropolis with more than 15 million population - in Northern China. Out- and inpatients were referred to the colonoscopy for any reason, including diagnosis, screening, surveillance as well as endoscopic treatment. All investigative protocols were approved by the ethic committee of TJMUGH; the clinical procedures were carried out in accordance with the Declaration of Helsinki.

The exclusion criteria were as follows: (1) subjects with insufficient data for analysis or refused to participate; (2) patients with a prior history of inflammatory bowel disease; (3) or a previous history of colectomy; (4) incomplete colonoscopy procedure; (5) poor bowel preparation. All participants were evaluated before enrollment. If an individual underwent more than one colonoscopies during the study period, all procedures were regarded as one examination.

### Colonoscopy procedure

All colonoscopies were performed by expert endoscopists at our endoscopy center. An electronic colonoscopy (CF-Q260, Olympus Optical Co., Tokyo, Japan) was used during the study period. Bowel preparation was achieved by using 2 L of polyethylene glycol the day before examination. The bowel preparation was rated as excellent, good, fair, or poor according to the degree of visibility of intestinal mucosa devoid of stool.

We regarded complete colonoscopy as cecal intubation followed by identification of ileo-cecal valve. The location of diverticulosis was defined as right-sided (involving the cecum, ascending colon, or transverse colon); left-sided (involving the splenic flexure, descending colon, or sigmoid colon); or bilateral (involving the entire colon). An instruction was sent to each endoscopist, which covering the purpose of our current study (learn more about the risk factors of uncomplicated colonic diverticulosis). In addition, our research staff were present during the examination when the gastroenterologists detected any diverticulum. The distribution, number and type of diverticula were documented in our digital database by the endoscopists.

### Baseline characteristics, risk factors and measurements

The current study was conducted by two well trained research staff (FY and YZ) in our department. After informed consent, all participants were asked to complete a detailed questionnaire through face-to-face interviews on the same day prior to colonoscopic examination or endoscopic treatment. The following information was collected in detail: demographics (including sex, age, educational level, body mass index [BMI]), and lifestyle factors (including exercise habit [<3 or ≥3 per week], red meat consuming [<100/d or ≥100/d], smoking index [defined as the duration of smoking years multiplied by the number of cigarettes per day, then was categorized into nonsmoker, <400 and ≥400], and current alcohol intake [defined as non-drinker, light/moderate drinker consuming 1–350 g of alcohol and heavy drinker at least 351 g per week]). Patients were also evaluated about comorbidities on account of sex differences regarding distinct spectrum of diseases. Notably, we evaluated the presence or absence of hypertension, diabetes mellitus, coronary heart disease, colonic polyps, upper gastrointestinal diseases (chronic/atrophic gastritis, reflux esophagitis and peptic ulcer), hepato-biliary diseases (cholelithiasis and fatty liver disease), dyslipidemia, thyroid diseases and rheumatologic diseases (rheumatoid arthritis and gout), etc. Prevalent hypertension, diabetes mellitus and dyslipidemia were confirmed in terms of self-reported diagnosis or taking specific medications.

Estimated regular use of prescription were NSAIDs, corticosteroid (prednisolone, methylprednisolone and dexamethasone), PPIs (esomeprazole, rabeprazole, omeprazole and lansoprazole), mucosal protective drugs (rebamipide, hydrotalcite and gefarnate), antihypertension drugs and hypoglycemic medications. We regarded regular use as continuously oral administration for at least 3 months (comprising NSAIDs, corticosteroid, PPIs and mucosal protective drugs) or 6 months (comprising antihypertension drugs and hypoglycemic medications) prior to our interview. We only took account of official prescription at our clinic or other certificated medical institution/pharmacy. Since it is difficult to definitely delineate, the usage of OTC, herbals and dietary supplementary were not recorded.

### Statistical analysis

Continuous data were demonstrated as mean (standard deviation) and categorical variables as counts or frequencies. In univariate analysis between binary groups according to the presence of diverticulosis, an independent student’s t test or the method of Mann-Whitney test was employed. Likewise, we compared categorical variables using Pearson’s χ^2^ or Fisher’s exact statistic to identify differences in the baseline characteristics. Age and BMI were evaluated by continuous variables. The risk factors for predicting the presence of diverticulosis were determined individually for men and women using a stepwise binary logistic regression model. We demonstrated odds ratio (OR) and 95% confidence interval (CI) for each independent variable. A *P* value < 0.05 was regarded as statistically significant and all tests were two-sided. The statistical analyses were performed with SPSS 22.0 (IBM, New York NY, USA) or STATA 12.0 (Stata Corporation, College Station Texas, USA).

### Availability of data and materials

Available under request.

### Consent for publication

The participant gave informed consent before taking part in this study. The samples were de-identified.

### Ethics approval and consent to participate

This study was conducted in accordance with the Declaration of Helsinki and was approved by Ethics Committee of Tianjin Medical University General Hospital.

## Electronic supplementary material

Table S1 Comparison of characteristics in whole study population
