# Supplementary material for: Sex differences in risk factors of uncomplicated colonic diverticulosis in a metropolitan area from Northern China
Source: Sci Rep. 2018 Jan 9;8:138. doi: 10.1038/s41598-017-18517-1 (PMC5760586; doi:10.1038/s41598-017-18517-1)
Supplement: Supplementary file 1 — Table S1 Comparison of characteristics in whole study population [file 41598_2017_18517_MOESM1_ESM.doc]

**Full Title**

Sex differences in risk factors of uncomplicated colonic diverticulosis in a metropolitan area from Northern China

**Running title**

Sex-related risk factors of diverticulosis

**Author names and Affiliations**

#Fang Yang^a, b^, M.M., #Yanmin Zheng^a, b^, M.M., Xihui Jiang^a, b^, M.M., Zhengyan Su^a, b^, M.M., Ya Wang^a, b^, M.M., Lin Lin^a, b^, M.M., Houning Lv^a, b^, M.M., Jie Zhang^a, b^, M.D., Ph.D., Jingwen Zhao^a, b^, Ph.D., Bangmao Wang^a, b^, M.D., Ph.D., Kui Jiang^a, b^, M.D., Ph.D. & Chao Sun^a, b^, M.D., Ph.D.

^a^ Department of Gastroenterology and Hepatology, Tianjin Medical University General Hospital, Anshan Road 154, Heping District, Tianjin 300052, China

^b^ Tianjin Institute of Digestive Disease, Tianjin Medical University General Hospital, Anshan Road 154,Heping District, Tianjin 300052, China

#These two authors contributed equally to this work.

**Correspondence address**

Kui Jiang, M.D., Ph.D.

E-mail: kjiang@tmu.edu.cn

Chao Sun, M.D., Ph.D.

E-mail: chaosun@tmu.edu.cn

Telephone: +86-022-60362608

Fax: +86-022-27813550

Table S1 Comparison of characteristics in whole study population

|  | Div (+) (n=218) | Div (-) (n=4,168) | *P* values |
| --- | --- | --- | --- |
| Sex  Men  Women | 148 (67.9)  70 (32.1) | 1,896 (45.5)  2,272 (54.5) | **< 0.001** |
| Age, (years), mean (SD)  Age (years), n (%)  ≤ 39  40-49  50-59  60-69  ≥ 70  Education (years), n (%)  ≤ 6  7-9  10-12  > 12  Residence, n (%)  Urban  Rural  BMI (kg/m^2^), mean (SD)  BMI, n (%)  < 25  25-30  > 30  Exercise habit, n (%)  ≤ 3 times/week  > 3 times/week  Red Meat, n (%)  < 100 g/d  ≥ 100 g/d  Smoking index, n (%)  Nonsmoker  < 400  ≥ 400  Alcohol consumption, n (%)  Non-drinker  Light/moderate drinker (1-350 g/week)  Heavy drinker (≥ 351 g/week)  Hypertension  Diabetes mellitus  Coronary heart disease  Colonic polyps  Upper gastrointestinal diseases  Hepato-biliary diseases  Dyslipidemia  Rheumatologic diseases  Miscellaneous  NSAIDs  Corticosteroid  PPIs  Mucosal protective drugs  Antihypertension medications  Hypoglycemic medications | 59.0 (11.0)  16 (7.3)  24 (11.0)  61 (28.0)  73 (33.5)  44 (20.2)  20 (9.2)  52 (23.9)  55 (25.2)  91 (41.7)  169 (77.5)  49 (22.5)  24.6 (3.5)  127 (58.3)  71 (32.5)  20 (9.2)  102 (46.8)  116 (53.2)  164 (75.2)  54 (24.8)  132(60.6)  31 (14.2)  55(25.2)  191 (87.6)  7 (3.2)  20 (9.2)  65 (29.8)  21 (9.6)  15 (6.9)  123 (56.4)  17 (7.8)  10 (4.6)  8 (3.7)  19 (8.7)  24 (11.0)  34 (15.6)  7(3.2)  16 (7.3)  59(27.1)  62(28.4)  22 (10.1) | 53.4 (13.7)  594 (14.3)  723 (17.3)  1,320 (31.7)  900(21.6)  631 (15.1)  349 (8.4)  1,014 (24.3)  1,053(25.3)  1,752 (42.0)  3,374 (81.0)  794 (19.0)  23.5 (3.3)  3,020 (72.4)  990(23.8)  158 (3.8)  2,036 (48.8)  2,132 (51.2)  3,568 (85.6)  600 (14.4)  3,344 (80.2)  275 (6.6)  549(13.2)  3,672 (88.1)  116 (2.8)  380 (9.1)  734 (17.6)  2,739(6.5)  190 (4.6)  1,826(43.8)  298(7.1)  168 (4.0)  102 (2.4)  70(1.7）  365 (8.8)  206 (4.9)  50 (1.2)  276 (6.6)  980 (23.5)  723 (17.3)  265 (6.4) | **< 0.001**  **< 0.001**  0.981  0.217  0.105  **< 0.001**  0.579  **< 0.001**  **< 0.001**  0.932  **< 0.001**  0.093  0.135  **< 0.001**  0.686  0.600  0.261  **<0.001**  0.270  **< 0.001**  **0.022**  0.675  0.252  **< 0.001**  **0.035** |

Data are expressed as mean ± SD or number (percentage)

Div (-): patients without diverticulosis

Div (+): patients with diverticulosis

*BMI* body mass index, *NSAIDs* non-steroidal anti-inflammatory drugs, *PPIs* proton pump inhibitors
